# Angst und Depression bei Jugendlichen

**DOI:** 10.1007/s00103-024-03849-x

**Published:** 2024-03-08

**Authors:** Franz Resch, Peter Parzer

**Affiliations:** Klinik für Kinder und Jugendpsychiatrie, Universitätsklinikum Heidelberg, Blumenstraße 8, 69115 Heidelberg, Deutschland

**Keywords:** Subklinische Depression, Angst und Depression gemischt, Scham, Emotionaler Dialog, Psychotherapie, Subclinical depression, Combination of anxiety and depression, Shame, Emotional dialogue, Psychotherapy

## Abstract

Ängste und Depressionen bei Jugendlichen haben schon in den Jahren vor der COVID-19-Pandemie zugenommen und dann im Pandemiegeschehen noch einmal eine deutliche Steigerung erfahren. In diesem Artikel werden die unterschiedlichen klinischen Ausdrucksformen dieser emotionalen Syndrome detailliert dargestellt und auch die Entwicklungswege einer Kombination beider Störungen expliziert. Auch subklinische Formen von Angst und Depression haben schon deutliche klinische Auswirkungen und beeinträchtigen die Entwicklungsaufgaben der Adoleszenz. Die „avolitionale Depression“ (Depression mit schweren Antriebsstörungen) wird als Sonderform erwähnt. Pathogenetische Bausteine – von einer genetischen Vulnerabilität bis zu psychosozialen Belastungsfaktoren – kommen im Licht der Tatsache zur Diskussion, dass Ängste und Depressionen beim weiblichen Geschlecht im Jugendalter etwa doppelt so häufig auftreten wie beim männlichen. Die Einbettung der Störungen in das aktuelle Zeitgeschehen zeigt die besondere Bedeutung der selbstreflexiven Emotion „Scham“ im jugendlichen Entwicklungsprozess. Vor einer Verknappung und Dysfunktionalität des emotionalen Dialogs zwischen wichtigen Bezugspersonen und Kindern muss gewarnt werden. Dessen Rolle für die Selbst- und Affektregulation der Jugendlichen ist nicht zu unterschätzen. Den Abschluss bildet eine Übersicht über die wichtigsten therapeutischen Maßnahmen bei Ängsten und Depressionen im Jugendalter.

## Abstract (en)

Anxiety and depression among young people had already increased in the years before the COVID-19 pandemic and then experienced a significant increase again during the pandemic. In this article the different clinical forms of expression of these emotional syndromes are presented in detail and the developmental paths of a combination of both disorders are also explained. Even subclinical forms of anxiety and depression already have clear clinical implications and impair the developmental tasks of adolescence. The “avolitional depression” (depression with severe drive disorders) is mentioned as a special form. Pathogenetic building blocks—from genetic vulnerability to psychosocial stressors—come up for discussion in light of the fact that anxiety and depression are about twice as common in adolescent females as in males. The embedding of the disorders in current events shows the special importance of the self-reflective emotion of shame in the adolescent development process. The scarcity and dysfunctionality of emotional dialogue between significant caregivers and children must be cautioned against. Its role in adolescents’ self-regulation and affect regulation should not be underestimated. Finally, an overview of the most important therapeutic measures for anxiety and depression in adolescence is presented.

## Einleitung

Ängste und Depressionen stellen die häufigsten psychischen Probleme im Jugendalter dar. Auch subklinische Formen können Jugendliche in ihrer Entwicklung beeinträchtigen. Die verschiedenen Bausteine der Pathogenese reichen von genetischer Vulnerabilität bis zu psychosozialen Risikofaktoren. In diesem Artikel sollen die Häufigkeitsunterschiede des Auftretens von Angst und Depression bei männlichen und weiblichen Jugendlichen diskutiert werden. Der Einbettung der Störungen ins aktuelle Zeitgeschehen ist ein eigener Abschnitt gewidmet. Da Angstgefühle und Verstimmungszustände sehr vielgestaltig und in ihrer Intensität sehr unterschiedlich sein können, sollen im Folgenden die Facetten dieser psychischen Probleme dargestellt werden, bevor ihre Genese diskutiert und auf das aktuelle Zeitgeschehen Bezug genommen wird.

## Facetten von Angst und Depression

### Ängste bei Jugendlichen

Angst ist ebenso wie Wut, Trauer oder Ekel eine der wichtigen Basisemotionen des Menschen. Angst kennzeichnet Bedrohungen, lässt uns auf der Hut sein und im Notfall die Flucht antreten. Angst löst eine innere Alarmreaktion aus, die sich zwar unangenehm anfühlt, aber durchaus für das Überleben wichtig sein kann. Zeiten der Unübersichtlichkeit, wie die COVID-19-Pandemie, können nicht nur Ängste, sich zu infizieren oder jemanden durch Krankheit zu verlieren, hervorrufen, sie sind auch Ausgangspunkt für Zukunftsängste, Angst vor Chaos und Sorgen um die Entwicklung der ganzen Familie.

Ab wann eine fürsorgliche Befürchtung, ab wann Ängste nicht mehr angemessen sind, bedarf einer definitorischen Festlegung. Ein Kriterium ist die Zeitdauer der Angst. Gefühle, die alarmieren und nicht desaktualisiert (zum Abklingen gebracht) werden können, erzeugen Belastungsdruck. Der Zeitraum der Persistenz wird hier bei Kindern und Jugendlichen oft kürzer gewählt als im Erwachsenenalter: beispielsweise bei der „Störung mit Trennungsangst“ 4 Wochen anstatt 6 Monaten im Erwachsenenalter. Ein zweites Kriterium zur Unterscheidung von normalen und pathologischen Ängsten ist die Intensität. Besonders heftige Gefühle, Betroffenheit und belastende begleitende Befürchtungen kennzeichnen die Angststörung. Auch die Angemessenheit des Gefühls in der auslösenden Situation kann herangezogen werden. Eine Angst, die ohne eine objektiv bedrohliche Situation auftritt, überschreitet die Grenze zur Störung. Eine Generalisierung der Auslöser, die praktisch den gesamten Alltag umfassen kann und zu ubiquitären Ängsten führt, ist schließlich immer pathologisch. Eine Interferenz mit den wichtigen Entwicklungsaufgaben und Lebensthemen der Jugendlichen kann deren Bewältigung verunmöglichen und dadurch negative Entwicklungsfolgen zeitigen.

Angststörungen sind die häufigsten Störungsmanifestationen im Jugendalter. Die weltweite Punktprävalenz bei Kindern und Jugendlichen vor der COVID-19-Pandemie wird in einer Metaanalyse von 41 Studien aus 27 Ländern mit 6,5 % (KI 95 %: 4,7–9,1 %) angegeben [1]. Etwa 8 % der Jugendlichen im Vereinigten Königreich (11–16 Jahre) erfüllten 2017 die Kriterien für eine Angststörung [2].

Störungen mit Trennungsangst (ICD-10^1^-Kodierung: F93.0), spezifische Phobien (F40.2), selektiver Mutismus (F94.0) und die soziale Angststörung (F40.10) beginnen in der Regel schon im Kindesalter, können aber durchaus in die Adoleszenz hineinreichen. Die Sorge der Trennungsangst, von den Eltern abgekoppelt, alleingelassen oder durch Verlust eines Elternteiles verwaist sein zu müssen, richtet sich direkt an wichtige Bezugspersonen. Anklammerungstendenzen prägen das Bild. Es handelt sich dabei um die häufigste Angst bei Kindern unter 12 Jahren, die im Kindesalter häufiger als im Jugendalter zu finden ist. Spezifische Phobien finden ihre Auslöser bei Tieren (z. B. Schlangen oder Spinnen), in unangenehmen Situationen (z. B. ärztlichen Untersuchungen und Blutabnahmen) oder allgemein in bedrohlich erlebten Umweltszenarien (z. B. Gewitter oder Dunkelheit). Auch Ängste vor Einbrechern oder Kriegsängste gehören in diese Kategorie, können aber bei Ausweitung von Befürchtungen und Sorgen auch in eine generalisierte Angststörung (s. unten) münden. Der Verlauf der Störung kann stark variieren. Der selektive Mutismus ist durch die Unfähigkeit gekennzeichnet, in bestimmten Situationen zu sprechen. Diese Sprechhemmung wird durch eine aktive Vermeidung hervorgerufen, wobei viele Betroffene sich durch nonverbale Mittel (z. B. schriftlich) in der Interaktion mit Erwachsenen oder fremden Menschen artikulieren. Es besteht ein enger Bezug zur sozialen Angst. Die soziale Angststörung ruft anhaltende Ängste in Situationen hervor, in denen sich Kinder oder Jugendliche vor anderen zeigen müssen. Es geht um die Themen Beurteilt-Werden, Beschämt- oder Verlacht-Werden, Sich-Blamieren, während man vor anderen eine Leistung zu erbringen hat. Die Befürchtung, beschämt zu werden, kann sich gegenüber Erwachsenen ebenso wie gegenüber Gleichaltrigen manifestieren. Diese Störung kommt bei Mädchen häufiger vor.

Die generalisierte Angststörung (F41.1) ist durch das Thema „Sorgen“ beherrscht. Die Betroffenen machen sich – aus der Sicht ihrer Umgebung – unbegründete, sinnlose und übertriebene Sorgen. Die furchtsame Erwartung, dass Unglück oder Krankheit ins Leben dieser Jugendlichen einbrechen könnten, kann noch durch die Angst, bestimmten Verpflichtungen im Alltag nicht gerecht zu werden, oder durch Verarmungsängste akzentuiert werden. Schlafstörungen, Konzentrationsprobleme und eine schwere Anspannung beherrschen das Bild. Die Agoraphobie (F40.0) ist eine übertriebene Angst vor Menschenansammlungen, bspw. in öffentlichen Verkehrsmitteln, Kinos oder im Theater. Das öffentliche Leben wird deswegen gemieden. Panikattacken (F41.0) sind anfallsartig auftretende Zustände plötzlicher intensiver Angst, die ohne Bezug auf eine spezifische Situation „aus heiterem Himmel“ die Jugendlichen heimsuchen und mit starken vegetativen Reaktionen einhergehen. Sie können Todesängste oder die Sorge, verrückt zu werden, auslösen.

Besonders hervorzuheben ist noch die posttraumatische Belastungsstörung (F43.1), die von einigen Autoren auch dem Spektrum der Angststörungen zugezählt wird [3]. Es handelt sich dabei um eine Folge von psychischen Reaktionen auf schwere seelische Traumatisierungen. Durch Intrusionen und in Tagträumen können Reste der traumatischen Situation immer wieder ins Tagesbewusstsein eindringen und Ängste hervorrufen. Vermeidungsverhalten, um eventuellen Auslösern der traumatischen Erinnerungen vorzubeugen, ist die Folge. Zusätzlich besteht eine gesteigerte Erregbarkeit. Solche Traumafolgestörungen sind subjektiv sehr belastend und können in Erschöpfungszustände münden.

Die Angststörungen sind in der Adoleszenz deshalb so bedeutend, weil sie eine stark negative Wirkung auf das psychosoziale Funktionsniveau der Jugendlichen ausüben [2]. Die Betroffenen haben intensive Vermeidungstendenzen, was ihr schulisches Fortkommen (z. B. durch Schulabsentismus) beeinträchtigen kann, auch in der Gleichaltrigengruppe erleiden diese Jugendlichen Nachteile, sie ziehen sich eher zurück, bleiben ausgeschlossen oder vereinsamen. Sie können die Aufgabe der Ablösung von der Familie nicht meistern und haben verstärkt Identitäts- und Selbstwertprobleme [4].

### Depressionen bei Jugendlichen

Depressionen im Jugendalter sind ebenfalls sehr häufig (geworden) und können sehr vielgestaltig sein. Eine Studie aus den USA [6] vor der COVID-19-Pandemie beschreibt eine Zunahme der 12-Monats-Prävalenz der klinisch signifikanten schweren Depression (Major Depression, Major Depressive Disorder – MDD, F32) zwischen 2011 und 2016 von 8,3 % auf 12,9 %. Die Entwicklungen im Rahmen der Pandemie und die deutlichen Geschlechtsdifferenzen zu Ungunsten von Mädchen (Depressionen zeigen sich in der Adoleszenz bei Mädchen etwa doppelt so häufig wie bei Jungen) sollen in einem eigenen Abschnitt besprochen werden (s. unten). Die MDD deckt sich in ihrer Symptomatik mit der in älteren Büchern beschriebenen „melancholischen Depression“. Müdigkeit und Energieverlust sind mit psychomotorischer Hemmung oder Unruhe verbunden. Gewichtsverlust (oder die Unfähigkeit, Gewicht zuzunehmen), Appetitverlust, Schlafstörungen (Ein- und Durchschlafstörungen oder Hypersomnie) werden durch deutliche Verstimmungssymptome ergänzt. Irritabilität, labile Stimmung mit Schwankungen oder Schweregefühl kennzeichnen das Bild. Traurigkeit und Weinen können in eine Art von verzweifelter „Versteinerung“ übergehen. Schuldgefühle, Schamgefühle, Gefühle der Wertlosigkeit, Hoffnungslosigkeit und Konzentrationsstörungen sowie Entscheidungsschwäche bis zur Entscheidungsunfähigkeit komplettieren den Symptomkanon. Häufig finden wir die Risikoverhaltensweisen der Suizidalität mit Todesgedanken, Todeswünschen bis hin zu konkreten Überlegungen, sich etwas anzutun, und elaborierten Suizidvorstellungen mit Planungscharakter. Bei US-amerikanischen Jugendlichen mit MDD beträgt die Häufigkeit von Suizidgedanken 30 %; 10,8 % hatten im letzten Jahr einen Suizidversuch gemacht [5]. Die Jugendlichen mit schweren depressiven Störungen erleiden einen deutlichen Funktionsverlust in Alltag und Schule [6].

Obwohl Jugendliche nach den Diagnosesystemen der Erwachsenen diagnostiziert werden, weicht ihr Symptombild signifikant von dem der Erwachsenen ab [7]. Adoleszente zeigen häufiger Irritabilität und eine labile Stimmung im Vergleich zu Erwachsenen, bei denen eine selektive Berührbarkeit nur für negativ getönte Ereignisse besteht. Darüber hinaus werden die vegetativen Symptome (Appetit- und Gewichtsveränderungen, Energieverlust und Schlafstörungen) von Jugendlichen häufiger präsentiert [7]. Auch somatische Beschwerden und ein sozialer Rückzug sind im Jugendalter häufiger zu beobachten [6].

Eine depressive und reizbare Verstimmung, die mindestens ein Jahr lang anhält, wird als Dysthymie oder persistierende depressive Störung (F34) bezeichnet. Appetitlosigkeit oder übermäßiges Essbedürfnis, Schlafstörungen oder mangelndes Schlafbedürfnis, Energieeinbuße und Erschöpfung, Konzentrationsstörungen, Entscheidungsschwäche und eine deutliche Hoffnungslosigkeit kennzeichnen dieses Symptombild, die Einzelsymptome erreichen aber nicht den Schweregrad der Major Depression. Die funktionellen Einschränkungen in Schule und Alltag können jedoch trotzdem signifikant sein. Risikoverhaltensweisen, wie nichtsuizidale Selbstverletzungen (NSSV), können das klinische Bild verkomplizieren.

Wenn Depressionen im Jugendalter beginnen, sind sie mit einem höheren Schweregrad späterer Depressionen im Erwachsenenalter verbunden. Es werden eine gesteigerte Zahl von Episoden, vermehrte Krankenhausaufenthalte und höhere Risiken der Selbstgefährdung durch Selbstverletzungen und Suizid berichtet (Übersicht bei [6]). Andere Autoren beschreiben erhöhte Risiken für Übergewicht, Diabetes und kardiovaskuläre Erkrankungen, was die Depression als chronische „Stresserkrankung“ kennzeichnet. Auch Beeinträchtigungen des sozialen und beruflichen Lebensweges werden berichtet [6].

### Angst und Depression gemischt

Komorbiditäten bei Angst und Depression sind häufig. So kommen bei über 60 % der depressiven Jugendlichen auch andere mentale Probleme hinzu. Die Odds Ratio für Angststörungen bei schweren Depressionen im Jugendalter (MDD) beträgt 3,96 (für Mädchen 4,09; für Jungen 3,73). Die Wechselwirkungen von Angst und Depression im Jugendalter scheinen aber viel komplexer, als es das simple Konstrukt einer „Komorbidität“ vermuten lässt. Immerhin findet sich das simultane Auftreten von Angst und Depression in 15–75 % der Fälle (Übersicht bei [8]). Da Ängste in einem früheren Kindesalter beginnen als Depressionen, spielen offenbar Temperamentfaktoren und Entwicklungsprozesse bei der gemeinsamen Entstehung eine Rolle. Hervorzuheben ist, dass Angst, die von Depression begleitet wird, klinisch gravierendere Verläufe aufweist als reine Angststörungen. Dies gilt aber nicht, wenn Depressionen die Hauptdiagnose bilden [8]. So zeigte sich, dass die Doppeldiagnose bei der Hauptdiagnose „Angststörung“ mit geringerem Funktionsniveau, stärkeren Problemen in den Familien, heftigeren Ängsten und massiveren Symptomen der Depression (z. B. negativer Stimmung, Anhedonie) verknüpft ist. Theoretische Modelle versuchen diese komplexen Zusammenhänge aus klinischer und pathogenetischer Sicht zu ordnen. Ein gemeinsamer Faktor wurde in dem Phänomen der negativen Affektivität gefunden. Es könnte sich dabei um einen transdiagnostischen Faktor handeln, der auch den Faktor „Neurotizismus“ und die Faktoren „Grübelneigung“ und „Intoleranz von Unsicherheit“ einschließt [8]. Das Modell von Cummings et al. [8] geht davon aus, dass Angst und Depression separate, aber miteinander in Beziehung stehende Konstrukte darstellen. In ihrem Modell weisen unterschiedliche Angststörungen unterschiedliche Arten der Komorbidität mit Depression auf. Es werden 3 Entwicklungswege unterschieden:

Entwicklungsweg 1 kennzeichnet Jugendliche mit einer starken Veranlagung für Ängste. Es handelt sich dabei um spezifische Temperamentfaktoren, wie z. B. behaviorale Inhibition, und um mögliche genetische Vulnerabilitäten. Aber auch beängstigende, einschüchternde Umwelten (z. B. in Familien) können eine Angstneigung erzeugen. Bestehende Ängste können wiederum mit starken negativen Affekten und einer besonderen Überempfindlichkeit gegenüber negativen Umwelteinflüssen die Entwicklung einer Depression bahnen. Typischerweise kommen für diesen Entwicklungsweg von der Angst in die Depression Trennungsängste und soziale Ängste in Betracht. Die Angst zeigt sich deutlich ausgeprägt, die Depression bleibt jedoch eher milde.

Entwicklungsweg 2 kennzeichnet Jugendliche, die gleichzeitig Angstgefühle und Depressionen aufgrund derselben Auslösesituation entwickeln. Angst und Depression sind deutlich, beide Störungen werden abwechselnd als im Vordergrund stehend betrachtet. Angst und Depression sind durch eine Überlappung ihrer Symptome gekennzeichnet: Die Jugendlichen machen sich Sorgen um Personen, sich selbst oder die Zukunft. Typischerweise handelt es sich um die Komorbidität von generalisierter Angst und Depression. Diese Gruppe weist offenbar die meisten geteilten Risikofaktoren (biologisch, psychisch, sozial) für beide Störungen auf. Familiäre und genetische Faktoren spielen dabei eine Rolle.

Entwicklungsweg 3 beschreibt Jugendliche mit einer primären Diathese für Depressionen, wobei die depressionsbezogenen Beeinträchtigungen, wie z. B. sozialer Rückzug, Schwierigkeiten im Umgang mit anderen Jugendlichen, Mobbing oder Isolation, schließlich zur Quelle von Ängsten werden. Dieser Entwicklungsweg betrifft typischerweise soziale Ängste und ebenfalls generalisierte Angststörungen. Interpersonale Risikofaktoren (Einsamkeit, maladaptive Elternschaft und Viktimisierungen) gelten für diese Art der Komorbidität als ausschlaggebend (für das gesamte Modell siehe [8]).

## Pathogenetische Bausteine

### Subklinische Syndrome

Das Spektrum von Angst und Depression im Jugendalter umfasst aber nicht nur die schweren, nosologisch definierten Störungsmuster, sondern ist durch einen unscharfen Rand von subklinischen Beschwerden vom normalen Empfinden getrennt. Diese Anpassungsstörungen mit ängstlicher oder depressiver Symptomatik (F43) sind auf bestimmte Auslöser bezogen und folgen diesen in zeitlichem Abstand oder sie sind unmittelbare Folgen traumatischer Ereignisse wie die posttraumatische Belastungsstörung. Depressive Verstimmungen mit Bedrücktheit und Trauerreaktionen, z. B. als Reaktion auf den Verlust einer wichtigen Bezugsperson, reichen tief in die Normalität als Bewältigungsversuch hinein. Es kommt zum Abzug von Bindungsbesetzungen (d. h., die emotionalen Bande zur verlorenen Bezugsperson werden gelockert), was eine Neuorientierung erst vorbereitet. Auch Angst vor unmittelbaren oder mittelbaren Bedrohungen und Gefahren ist eine durchaus normale Reaktion. Die Persistenz solcher Stimmungsveränderungen und Befürchtungen über einen längeren Zeitraum beginnt schließlich aber mit der kindlichen/jugendlichen Entwicklung zu interferieren. Halten solche Zeichen von Distress und Besorgtheit längere Zeit an, besteht das Phänomen einer subklinischen oder unterschwelligen Depression (Subthreshold Depression; [9]) oder einer subliminalen (von anderen oft gar nicht wahrnehmbaren) Angstsymptomatik. Es ist daher sinnvoll, diese Störungen nicht nur kategorial zu diagnostizieren, sondern auch dimensional als Beschwerden zu erfassen. Die klinische Bedeutsamkeit und Entwicklungsrelevanz der unterschwelligen Depression konnten klar bestätigt werden [10]. So war diese Depressionsform ganz ähnlich wie die Major Depression bei Mädchen häufiger, bewirkte ebenfalls deutliche funktionelle Einschränkungen, hatte eine ähnliche Komorbidität und führte ebenfalls zur Inanspruchnahme therapeutischer Hilfen. Auch die Suizidalität, Selbstverletzungstendenzen, Hirnfunktionsstörungen (Verringerungen der Hippocampusdicke) und das genetische Belastungsmuster (familiäre Häufungen) zeigten sich in ähnlicher Form [10]. Unterschwellige Depressionen bei Jugendlichen führen in über der Hälfte der Fälle zu depressiven Vollbildern im Erwachsenenalter [10].

Angst und Depression können direkt als reaktive Symptome auf Belastungen entstehen. Sie betreffen bspw. Jugendliche mit körperlichen Erkrankungen. So ist ein Diabetes Typ 1 mit einer erhöhten Prävalenz von Ängsten und Depressionen verbunden, was zu Problemen des therapeutischen Diabetesmanagements führen kann [11]. Auch bei Überlebenden von Krebserkrankungen im Erwachsenenalter finden sich Ängste und Depressionen gehäuft [12]. Kindliche Traumatisierungen, die mit körperlichen Misshandlungen und sexuellen Grenzüberschreitungen einhergehen, führen mit erhöhter Wahrscheinlichkeit zu Angst, Depression und posttraumatischen Stressstörungen bei jungen Erwachsenen [13]. Unterschiedliche Traumata in unterschiedlichen Lebensaltern und mit unterschiedlicher Dauer führen zu differenzierten Störungsmustern im späteren Lebensalter [13].

Während Ängste einer Überreaktion auf Umweltstimuli entsprechen, scheint die Depression eine chronifizierte Störung der Stressregulation zu repräsentieren. Psychische Störungen sind nur durch eine Wechselwirkung multipler Risikofaktoren mit dem Entwicklungsprozess der Adoleszenz zu erklären [14]. Etwa 40 % der Varianz der Depression lassen sich durch genetische Faktoren interpretieren [9]. Ein polygenetisches Anlagerisiko ist anzunehmen. Dieses genetische Risiko scheint bei rezidivierender Depression, starker Symptomausprägung und früh beginnenden Depressionen in erhöhtem Maße vorzuliegen [9]. Auch der Übergang in eine bipolare Störung mit hypomanischen und manischen Phasen erscheint genetisch getriggert und kommt in Familien mit einer Geschichte von bipolaren Störungen gehäuft vor [6]. Unbestreitbar ist, dass die genetische Vulnerabilität – die vielleicht in Temperamentsfaktoren wie behavioraler Inhibition (Hemmung gegenüber Unbekanntem oder Überraschendem) oder einer Präponderanz (Vorherrschen und Überwiegen) negativer Affekte zum Ausdruck kommt – erst in einer Wechselwirkung mit psychosozialen Stressoren zur Krankheit führt. Als Risikofaktoren werden chronische somatische Krankheiten, verstärkte Irritabilität und vor allem kindliche Ängste benannt. Frühe Misshandlungen, Vernachlässigungen und Störungen der Eltern-Kind-Interaktion (z. B. bei elterlichen psychischen Störungen) gelten ebenfalls als ungünstige Mediatoren. Auslöser der ersten Symptomatik können vielfältige psychosoziale Stressoren sein. Akute Belastungen sind nicht selten chronischen Dauerbelastungen aufgepfropft. Diese Belastungen können in Form von Vernachlässigungen, Gewalterfahrungen oder sexuellen Grenzüberschreitungen in den Familien, aber auch im Umfeld (dem schulischen Alltag, der Nachbarschaft) wirksam werden. Verluste von wichtigen Bezugspersonen sind bedeutsame Risikofaktoren. Immer noch wird im Schulalter das Mobbing unterschätzt [15].

Die verminderte Fähigkeit, negative Affekte zu kontrollieren und zu regulieren, muss nicht nur eine genetische Ursache besitzen. Kinder lernen mithilfe ihrer wichtigen Bezugspersonen, in einem Prozess der „sozialen Referenzierung“, ihre Gefühle besser zu verstehen und zu desaktualisieren. Dieser Prozess wird auch Co-Regulation genannt. Bei Störungen der Interaktion mit den Bezugspersonen bleibt diese Co-Regulation aus, die kindlichen Regulationsmöglichkeiten können bei belastenden Umwelten in der Frühzeit überfordert werden, sodass vernachlässigte und misshandelte Kinder vermehrt ihren negativen Gefühlen ohne Regulationskompetenz ausgeliefert sind. Das kann eine Risikospirale in Richtung Angst und Depression in Gang setzen [14].

### Risikoverhaltensweisen, Angst und Depression

Ängste und Depressionen sind als psychische Beschwerdebilder im Jugendalter eng mit Verhaltensweisen verbunden, die als Risikoverhaltensweisen bezeichnet werden. Es handelt sich dabei bspw. um Substanzmissbrauch (der neben Experimentierfreude und Rauscherleben auch im Sinne von Selbstberuhigung und Selbstmedikation zur Erleichterung von Symptomen verstanden werden kann), suchtartigen Internetkonsum, Selbstverletzungstendenzen und/oder exzessives Diätverhalten. Risikoverhalten stellt nicht per se eine psychische Störung dar, es ist Ausdruck einer besonderen Lebensstilbildung, es wird gewählt, bewusst eingesetzt, aktiv herbeigeführt und dient in der Regel der Stiftung von Identitätsgefühlen, einer Stabilisierung des Selbstwertes und einer Dokumentation von Zugehörigkeit [14]. Während Risikoverhaltensweisen also eher das Ziel haben, Grenzen auszutesten, den subjektiven Spielraum zu erweitern und eine persönliche Stärkung von Selbstwert und Identität zu erfahren (also durchaus auch einer Symptomabwehr dienen können), besitzen Angst und Depression immer einen Leidenscharakter, der in den Symptomen zum Ausdruck kommt. Oft begegnet uns bei Jugendlichen aber die Fassade des Risikoverhaltens, hinter der die Ängste und eine Verzweiflung erst indirekt fassbar werden. Über die Beziehung von Risikoverhalten und psychopathologischen Symptomen konnte eine internationale Arbeitsgruppe [16] folgende Zusammenhänge herausarbeiten: Die Studie umfasste die Daten von über 11.000 Jugendlichen in ganz Europa. Es zeigte sich, dass riskanter (in Zeit und Intensität aber noch kompensierter) Internetkonsum signifikant stärker mit anderen Risikoverhaltensweisen verknüpft war als mit psychopathologischen Symptomen. Demgegenüber zeigten Jugendliche mit suchtartigem pathologischen Internetkonsum einen deutlichen statistisch signifikanten Zusammenhang mit Depression, Angst und anderen Verhaltensauffälligkeiten. Zusammenhänge mit anderen Risikoverhaltensweisen standen eher im Hintergrund [16].

Risikoverhaltensweisen besitzen einen grundlegenden funktionalen Aspekt. Sie dienen dem jugendlichen Selbst und seiner Regulierung [14]. Sie besitzen aber für die gesundheitliche und soziale Entwicklung der Jugendlichen ein hohes Gefahrenpotenzial. Eine große Metaanalyse [17] bestätigte an 11 Studien mit 23.317 Adoleszenten, dass Konsumenten von Cannabis ein erhöhtes Risiko für die Entwicklung von Depressionen im jungen Erwachsenenalter aufweisen. Zusammenhänge mit einer Entwicklung von Angststörungen konnten nicht nachgewiesen werden. Risikoverhaltensweisen können Vorläufer und Begleiter psychischer Störungen – im Besonderen von Ängsten und Depressionen – sein, auch wenn ihr Ausgangspunkt immer ein Lebensstilverhalten ist, das eine Reaktion auf die aktuelle Umwelt darstellt.

### Die avolitionale Depression

Depressionen mit schweren Antriebshemmungen kennzeichnen einen Typ von Depression, der im klinischen Feld zunehmend zur Beobachtung gekommen ist. Es handelt sich dabei um Zustandsbilder mangelnder Aktivität, insbesondere zielgerichtete Handlungen werden unterlassen. Planlosigkeit und Untätigkeit sind aber nicht mit genussvoller Faulheit zu verwechseln, es handelt sich um schmerzvolle innere Blockaden, die mit verringertem Selbstwert, Überforderungsgefühlen und der Angst zu versagen einhergehen. Ein zentraler Makel des „ich bin nicht gut genug“ lässt die Patienten untätig zurück. In den Kliniken sehen wir diese Form der Depression gehäuft bei männlichen Individuen. Sie liegen tagsüber im Bett, haben keine Energie aufzustehen, können sich zwischen Alternativen nicht entscheiden, gerade eine Vielfalt von Möglichkeiten oder Angeboten scheint sie besonders zu lähmen. Termine werden verschleppt und nicht eingehalten, die Betroffenen können Verpflichtungen nicht nachkommen, ziehen sich zurück und schränken ihre Kommunikation ein. Es findet sich eine Angst, sich zu blamieren, Fehler zu machen, zu versagen. Ein zentrales Gefühl stellt die Scham dar; Kränkungen und Beschämungen können schon durch minimale Kritik oder allein das Ansprechen der Untätigkeit hervorgerufen werden. Manche Autoren versuchen für diese Patientengruppe das Konzept der narzisstischen Depression heranzuziehen [18]. Eine Untersuchung an außerfamiliär (z. B. in Heimen) untergebrachten Jugendlichen in Portugal zeigte gesteigerte Raten von Depressivität bei diesen Jugendlichen. Zusammenhänge zwischen mangelnden Erfahrungen der emotionalen Wärme und Geborgenheit waren mit verstärkter Schamneigung verknüpft, diese wiederum zeigte sich signifikant mit erhöhter Selbstkritik verbunden. Die Depression wies schließlich einen statistisch hochsignifikanten Zusammenhang mit Selbstkritik auf [19]. Jugendliche mit einer Neigung zu Scham scheinen besonders sensibel auf Kritik und Misserfolge zu reagieren [20]. Eine Metaanalyse von Studien zur Bedeutung früher Schemata für die Entwicklung von Depressivität bis ins Erwachsenenalter kommt zu dem Schluss, dass Individuen, die das Gefühl haben, nicht dazu zu gehören, sondern fehlerhaft, schlecht oder nicht liebenswert zu sein, höhere Depressionswerte aufwiesen. Soziale Isolation und Scham stellen offenbar wichtige Mediatoren dar [21].

## Epidemiologische Entwicklungslinien und Geschlechtsdifferenzen

Geschlechtsdifferenzen in der Präsentation von Ängsten und depressiven Symptomen bei Jugendlichen sind wiederholt bestätigt worden. Es ist davon auszugehen, dass Mädchen etwa doppelt so häufig an diesen emotionalen Symptomen leiden als männliche Jugendliche [22]. Eine globale Übersicht über die Entwicklung der Prävalenzzahlen vor und nach der COVID-19-Pandemie kommt zu folgenden Ergebnissen: Die Punktprävalenz an erhöhten Depressionswerten lag im Zeitraum 2001 bis 2010 bei etwa 24 % und steigerte sich im Zeitraum 2011 bis 2020 auf 37 % [23]. Weibliche Jugendliche zeigten auch in diesen Steigerungsraten eine erhöhte Prävalenz gegenüber den männlichen. Die gepoolte Lebenszeitprävalenz für Major Depression (MDD), die über alle Studien und den gesamten Zeitraum bei allen Geschlechtern gemeinsam errechnet wurde, lag um 19 %.

Die Erklärungen, warum Mädchen mehr internalisierende Symptome aufweisen als Jungs, sind dürftig und reichen von biologischen Differenzen (genetisch und hormonell) zu psychologischen Faktoren wie Geschlechtsrollenstereotypen [23]. Mädchen sind aber auf ihrem Weg, die jugendlichen Entwicklungsaufgaben zu meistern und den gesellschaftlichen Forderungen zu entsprechen, vermehrt Stressoren ausgesetzt [24]. Die Auswirkungen schwerer Traumata sind bei beiden Geschlechtern nicht signifikant unterschieden [25].

Die Metaanalyse einer kanadischen Arbeitsgruppe schloss weltweit 191 Studien ein, die an über 1,3 Mio. Jugendlichen durchgeführt worden waren. Sie kommt zu dem Schluss, dass die gepoolte Prävalenz von depressiven Symptomen (31 %) und Ängsten (31 %) deutlich angestiegen ist. Wieder sind Mädchen signifikant häufiger betroffen [26].

Eine deutsche Studie zu Ängsten und Lebensqualität von Jugendlichen vor und nach der Pandemie ergab, dass die gesundheitsbezogene Lebensqualität sich mit erniedrigten Werten bei 40,2 % (gegenüber 15,3 % vor der Pandemie) deutlich verschlechtert hatte. Erhöhte Angstwerte stiegen von 14,9 % in der Pandemie auf 24,1 % an [27].

Nach der COVID-19-Pandemie fanden in Deutschland 19 % aller Krankenhausbehandlungen bei Jugendlichen im Alter von 10–17 Jahren wegen psychischer Erkrankungen statt. Der häufigste Grund waren depressive Verstimmungen. Im Jahr 2021 waren psychische Erkrankungen und Verhaltensstörungen die häufigste Ursache für Krankenhausbehandlungen von Jugendlichen, 80.965 Jugendliche waren davon betroffen [28]. Den häufigsten Behandlungsgrund stellten mit 21.916 Fällen die Depressionen dar. Der Behandlungsanteil von psychischen Erkrankungen bei Krankenhausaufenthalten steigt seit 2011 kontinuierlich an. Dieser Anstieg war also schon vor der Pandemie zu verzeichnen [28].

Wir müssen anhand der uns vorliegenden Zahlen davon ausgehen, dass ein schon vor der Pandemie nachweisbarer Anstieg der Prävalenzen psychischer Störungen [29, 30] sich in der COVID-19-Pandemie noch deutlich verstärkt hat.

### Wie sind diese Steigerungen der Prävalenzraten zu verstehen?

Die psychischen Folgen der Pandemie haben sich bei Kindern und Jugendlichen mit sozioökonomischen Risiken, Migrationshintergrund und beengten Lebensverhältnissen signifikant schwerwiegender ausgewirkt [27]. Jugendliche mussten ihren Lebensstil radikal verändern, der Kontakt zu Peers war unterbunden (außer via soziale Medien). Die Regelmäßigkeit von Schule und Ausbildung war aufgelöst, Jugendliche, die vor der Pandemie schon ängstlich waren, erlebten verstärkte Sorgen und Befürchtungen bis in ein klinisches Ausmaß. Einsamkeit, soziale Distanzierung und die Notwendigkeit, durch einen erhöhten Internetkonsum die soziale Isolation zu überwinden, zeigten ebenfalls Wirkungen [31].

Mit genetischen Vulnerabilitäten allein sind diese Prävalenzanstiege nicht zu erklären. Es sind offenbar die gesteigerten psychosozialen Stressoren, die auch bei weniger prädisponierten Menschen schließlich doch die Symptomschwelle überschreiten.

## Einordnung in das aktuelle Zeitgeschehen

### Angst, Depression und Zeitgeist

Unsere spätmoderne Lebenswelt wird immer wieder nicht nur mit positiven Aspekten der Mannigfaltigkeit, des Wandels und der kulturellen Erweiterung, sondern auch mit negativen Aspekten der Beliebigkeit, der Verwirrung, der Manipulation und mangelnden Überschaubarkeit in Verbindung gebracht. Es fällt jungen Menschen nicht immer leicht, in Vielfalt und Mehrdeutigkeit ihre Orientierung zu finden. In den letzten Jahren sind neben der drastischen Pandemie noch neue Aspekte des Krieges, des Terrors, der Inflation, also der Kriegs- und Existenzangst, bis an unsere Haustüren gerückt. Ab welchem Zeitpunkt beginnen Zukunftsängste und Existenzsorgen die Jugendlichen in ihren Entwicklungsbestrebungen zu lähmen? Ab welchem Grad der Vervielfältigung von Möglichkeiten beginnt eine Agonie der Wahl? Ein Pluralitätsgewinn wird durch die zunehmende Verkomplizierung des Alltags wettgemacht. Wo Skepsis und Ängste beginnen, beginnen auch Verschwörungserzählungen und „ewige Wahrheiten“ mit ihren Welterklärungen. Wo Komplexität verwirrt, beginnen Glaube und Ideologie einfache Ordnungen zu versprechen. Auch eine reaktionäre Bodenständigkeit sucht nach einfachen Lösungen des Dilemmas. Woher nimmt die Jugend ihre Zukunftshoffnung? Wird in der lauten Welt der Konflikte die Stimme der Jugend überhaupt wahrgenommen? Sind nicht Egoismus, Wettbewerb, Leistungsdruck und Konsumhaltungen längst in den Mikrostrukturen der Familie angekommen [32]? Es liegt auf vielen Jugendlichen eine Last, etwas Besonderes hervorbringen zu müssen [14].

### Die Bedeutung der Scham im kulturellen Kontext

Um sich in der Erwachsenenwelt gut zu positionieren, brauchen Jugendliche eine hohe Qualität ihrer Ausbildung, eine gute Selbstreflexion und Selbstregulation sowie gute kommunikative Fähigkeiten, um sich aussichtsreich präsentieren zu können. Byung-Chul Han [33] spricht ja schon länger von einem Hamsterrad der narzisstischen Selbstausbeutung auf dem Weg zum persönlichen Erfolg jedes Einzelnen. Dabei haben sich die Bedingungen eines möglichen Scheiterns gewandelt. Den jungen Menschen wird suggeriert, dass ihnen alle Chancen offenstehen, allen Horrornachrichten zum Trotz. Aber dieses Angebot ist eine Illusion. Wo in der Nachkriegszeit noch die vorherrschenden Verengungen einen neurotischen Überbau des „ich darf nicht“ konstruierten, ist das persönliche Scheitern heute nicht mehr äußeren Grenzen oder sozialen Netzwerken geschuldet, sondern ausschließlich der eigenen Unfähigkeit zuzuschreiben. Das allgemeine „es geht halt nicht“ ist einem demütigenden „ich kann nicht“ gewichen. Das Gefühl, nicht gut genug zu sein, minderwertig zu sein, leistet einem beschämten Rückzug Vorschub. Der zentrale Affekt des Scheiterns ist heute die Scham [14].

### Der gefährdete emotionale Dialog – eine begründete Hypothese

Zwischen dem Auftreten von Angst und Depression bei Jugendlichen und dem familiären Klima gibt es signifikante Zusammenhänge. Ein systematischer Review über elterliche Faktoren ergab, dass das Risiko für Angst und Depression bei Jugendlichen mit mangelnder emotionaler Wärme, verstärkten Konflikten zwischen den Eltern, Überinvolviertheit in Erziehungsfragen oder emotionaler Ablehnung signifikant verknüpft war [34]. Eine eigene Studie an Jugendlichen mit Selbstverletzungen und Symptomen eines Borderlinesyndroms zeigte ebenfalls, dass mütterliche Vernachlässigung und eine gestörte Familienfunktion neben sexueller Traumatisierung einen Erklärungswert für das jugendliche Risikoverhalten besaß [35].

Nimmt man diese Fakten ernst und setzt sie in Beziehung zu den gesellschaftlichen Entwicklungen, scheint sich eine gefährliche Schere aufzutun. Während ein hohes Maß an Selbstregulation und emotionaler Kompetenz von den Jugendlichen im Prozess des Erwachsenwerdens gefordert wird, werden Eltern zunehmend durch die gesellschaftlichen Belastungen ökonomisch, arbeitstechnisch, in Freizeitkultur und Alltag unter Druck gebracht und von ihrer Beziehungs- und Erziehungsarbeit mit den Kindern abgelenkt. Verknappen Eltern den emotionalen Dialog und die Chancen einer Co-Regulation mit ihren Kindern? Zeitmangel, Ungeduld, Erschöpfung, Missverständnisse und eigene Orientierungslosigkeit fließen in den emotionalen Dialog und seine Qualität mit ein [14]. Wenn Eltern selbst bereits psychische Störungen aufweisen, werden Kinder in eine dysfunktionale Parentifizierung gezwungen oder früh in eine vernachlässigende Unabhängigkeit entlassen. Andere werden narzisstisch besetzt und in eine erfolgsorientierte Erziehungsstruktur mit Leistungsdruck gezwängt. Ein gestörter und/oder beeinträchtigter emotionaler Dialog kann sich aber fatal auf die emotionale Selbstregulation, Identitätsbildung und Selbstwertstabilisierung bei Jugendlichen auswirken. Jugendliche sind dann nicht in der Lage, den komplexen gesellschaftlichen Herausforderungen nachzukommen. Risikoverhaltensweisen und emotionale Probleme können die Folge sein. Zunehmende Störungen des emotionalen Dialogs wären somit an einer Zunahme von Symptombildungen im Jugendalter beteiligt [14].

## Therapeutische Ansätze und die Versorgungsrealität

Jede Form einer therapeutischen Intervention basiert auf einer adäquaten Diagnostik, die auch die Familie mit einbeziehen sollte. Einen Überblick über diagnostische Instrumente gibt [9]. Dabei ist besonders wichtig auch das Ausmaß an Suizidalität zu erfassen und angemessen auf diese Gefahr zu reagieren. Für Ängste und Depressionen werden multimodale Interventionen empfohlen, die mit psychoedukativen Beratungen der Adoleszenten und ihrer Angehörigen beginnen [6]. In den ersten Beratungen sollen die Rahmenbedingungen, Risikofaktoren und Gefährdungen so erörtert werden, dass ein gemeinsames Verständnis der Störung möglich wird.

Therapeutische Interventionen schließen Veränderungen des Lebensstils und eine Psychotherapie in erster Linie mit ein. Es geht um Aktivierungen [36], körperliche Betätigung, gesunde Ernährung und ausreichend Schlaf. Vermeidungshaltungen sollen angesprochen und schließlich in der Psychotherapie bearbeitet werden. Neben einer kognitiv-behavioralen Therapie [6] haben sich zur Depressionsbehandlung auch andere psychodynamische Interventionen oder Therapieformen der dritten Welle der Verhaltenstherapie bewährt [37]. Dazu gehören die „Achtsamkeitsverfahren“ und die „Akzeptanz- und Commitmenttherapie“ (ACT). Während die deutschen Therapieleitlinien derzeit veraltet und in Überarbeitung sind, kann auf internationale Leitlinien Bezug genommen werden [6]. In der Behandlung von Ängsten ist ebenfalls die Psychotherapie als primäre Therapieform hervorzuheben [2].

Die Einbeziehung der Familie in die Behandlung von Depressionen und Ängsten wird empfohlen, bei Depressionen scheint dadurch eine Wirksamkeitsverbesserung der Interventionen möglich zu sein [38].

Die Entscheidung, eine medikamentöse Behandlung mit Antidepressiva zu beginnen, sollte im Therapieprozess als kollaborativer Entschluss zwischen Therapeuten, Eltern und Patienten gefällt werden. Risiken und Gefahren sind dabei abzuwägen. Auf Einzelheiten der medikamentösen Behandlung kann hier nicht eingegangen werden, wobei selektive Serotonin-Wiederaufnahmehemmer (SSRIs) in den Leitlinien als Antidepressivum empfohlen werden (Übersicht bei [6]).

Weitere Interventionen, wie z. B. transkranielle Magnetstimulation, sind noch in einem experimentellen Stadium und können nicht als Routinetherapie empfohlen werden [39]. Verbesserte Versorgungsangebote am Übergang vom Jugendalter zum Erwachsenenalter sind als spezifische Transitionsangebote derzeit noch ein Desiderat [40].

Alle therapeutischen Interventionen bei Depression und Angst im Jugendalter bewegen sich in einem Spannungsfeld zwischen Wünschen nach Optimierung psychischer Funktionen (Enhancement) und Förderungen eigener Initiativen der Betroffenen (Emanzipation). Das therapeutische Ziel ist es, mehr Freiheitsgrade für persönliches Handeln bei den Jugendlichen zu erzielen. Nur selbst verwirklichtes Wissen – nicht bloß gelernte Erklärungen – können Einstellungen und Haltungen von Jugendlichen so verändern, dass sie ihr Verhalten ändern [14].

## Fazit

Ängste und Depressionen stellen die häufigsten psychischen Komplikationen im Jugendalter dar. Sie haben in den letzten Jahren eine Prävalenzerhöhung erfahren, die versorgungsrelevant ist. Da Angstgefühle und Verstimmungszustände sehr vielgestaltig und in ihrer Intensität sehr unterschiedlich sein können, wurden die unterschiedlichen Facetten dieser psychischen Probleme dargestellt, ihre Risikofaktoren und pathogenetischen Entwicklungswege diskutiert und ihre Einbettung in das aktuelle Zeitgeschehen untersucht. Ängste und Depressionen gefährden die jugendliche Entwicklung. Sie können jedoch erfolgreich durch eine multimodale Therapie behandelt werden.

## Footnotes

1 ICD-10: 10. Version der internationalen statistischen Klassifikation der Krankheiten und verwandter Gesundheitsprobleme.
